# Effect of HIV/HAART and Other Clinical Variables on the Oral Mycobiome Using Multivariate Analyses

**DOI:** 10.1128/mBio.00294-21

**Published:** 2021-03-23

**Authors:** Paul L. Fidel, Zach A. Thompson, Elizabeth A. Lilly, Carolina Granada, Kelly Treas, Kenneth R. Dubois, Laura Cook, Shahr B. Hashmi, Daniel J. Lisko, Chiranjit Mukherjee, Jose A. Vazquez, Michael E. Hagensee, Ann L. Griffen, Eugene J. Leys, Clifford J. Beall

**Affiliations:** a Center of Excellence in Oral and Craniofacial Biology, Louisiana State University Health Sciences Center School of Dentistry, New Orleans, Louisiana, USA; b Division of Pediatric Dentistry, The Ohio State University College of Dentistry, Columbus, Ohio, USA; c Division of Biosciences, The Ohio State University College of Dentistry, Columbus, Ohio, USA; d Department of Medicine, University of British Columbia, Vancouver, British Columbia, Canada; e Division of Infectious Diseases, Department of Medicine, Augusta University, Medical College of Georgia, Augusta, Georgia, USA; f Section of Infectious Disease, Department of Medicine, Louisiana State University Health Sciences Center, New Orleans, Louisiana, USA; University of Michigan Medical School

**Keywords:** oral microbiome, *Candida*, *Malassezia*, fungal microbiome, mycobiome, clinical variables, human immunodeficiency virus

## Abstract

The oral microbiome is considered an important factor in health and disease. We recently reported significant effects of HIV and several other clinical variables on the oral bacterial communities in a large cohort of HIV-positive and -negative individuals. The purpose of the present study was to similarly analyze the oral mycobiome in the same cohort. To identify fungi, the internal transcribed spacer 2 (ITS2) of the fungal rRNA genes was sequenced using oral rinse samples from 149 HIV-positive and 88 HIV-negative subjects that had previously undergone bacterial amplicon sequencing. Quantitative PCR was performed for total fungal content and total bacterial content. Interestingly, samples often showed predominance of a single fungal species with four major clusters predominated by Candida albicans, Candida dubliniensis, Malassezia restricta, or Saccharomyces cerevisiae. Quantitative PCR analysis showed the *Candida*-dominated sample clusters had significantly higher total fungal abundance than the *Malassezia* or *Saccharomyces* species. Of the 25 clinical variables evaluated for potential influences on the oral mycobiome, significant effects were associated with caries status, geographical site of sampling, sex, HIV under highly active antiretroviral therapy (HAART), and missing teeth, in rank order of statistical significance. Investigating specific interactions between fungi and bacteria in the samples often showed *Candida* species positively correlated with *Firmicutes* or *Actinobacteria* and negatively correlated with *Fusobacteria*, *Proteobacteria*, and *Bacteroidetes*. Our data suggest that the oral mycobiome, while diverse, is often dominated by a limited number of species per individual; is affected by several clinical variables, including HIV positivity and HAART; and shows genera-specific associations with bacterial groups.

## Abstract (precis)

The oral microbiome is likely a key element of homeostasis in the oral cavity. With >600 bacterial species and >160 fungal species comprising the oral microbiome, influences on its composition can have an impact on both local and systemic health.

## INTRODUCTION

HIV disease continues to be an important infection worldwide, with impacts on health and the economy. Persons infected with HIV have been particularly vulnerable to a variety of oral diseases, including gingivitis, periodontitis, dental caries, endodontic infections, oropharyngeal candidiasis (OPC), oral warts, oral hairy leukoplakia, and Kaposi’s sarcoma (1–3). However, over the past decade, advances in highly active antiretroviral therapy (HAART) as well as new guidelines and the earlier initiation of treatment have had a substantial impact on the HIV-positive population in the United States, manifested by a reduction in opportunistic infections/diseases, improved overall health, and a better quality of life (1). However, the fact that HAART has not been available or provided worldwide has left certain geographical areas with continued high rates of HIV-related oral manifestations (4, 5).

The oral microbiome is considered an important factor in health and disease. For example, several studies have shown changes in the composition and diversity of the oral microbiome during periodontal disease (6–8) and dental caries (6, 9). These studies have focused primarily on the bacterial microbiome. There are likely to be cross influences between host immunity and several types of endogenous microbiota. HIV infection and the resulting reduction in CD4 T cells can disrupt this balance, which in turn can influence susceptibility to oral opportunistic infections. Accordingly, community shifts of the oral microbiome similar to those seen in periodontal disease and caries could occur with HIV infection or the use of HAART. As such, the oral microbiome may be an effective therapeutic target. At present, studies characterizing the oral microbiome in HIV disease are in their infancy, with data derived primarily from small sample size studies (10–14). Results, including a recent report from our group that evaluated a large sample size, describe a modest, yet consistently significant effect of HIV on the overall composition of the oral bacterial microbiota.

The fungal community (mycobiome) represents another prominent component of the oral microbiome. The mycobiome has been much less studied than the bacterial community at any anatomical site, including the oral cavity. However, published studies show a more limited fungal representation in the oral cavity, with suggestions of possible antagonism leading to dominance of genera/species (15–20). These finding imply interesting interactions/associations within the mycobiome and between the mycobiome and bacterial community (15, 18, 21, 22). To date, there are only a few oral mycobiome studies conducted in HIV-positive subjects. These studies have relatively small sample sizes with varied results (10, 22–25).

There are a multitude of clinical factors or conditions that could be potential modulators of the oral microbiome and thus are considered confounders when examining the effects of HAART in HIV infection. Examples include variables such as antimicrobials; other prescribed medications; oral hygiene practices, including dental cleaning; cigarette smoking; xerostomia; diet; beverage intake, including alcohol; illicit drug use; periodontal disease; gingivitis; caries status and restorations; or remaining teeth. While a limited number of variables have been evaluated as an influence on the oral microbiome in previous studies of HIV-positive persons (i.e., viral load, CD4 cells, periodontal disease, and smoking), (10, 26–29), they have only been analyzed independently. We recently reported, using a large cohort with adequate balance and multivariate analyses, that several of these clinical variables had a significant influence on the oral bacterial community (30).

The purpose of the present study was to characterize the oral mycobiome in our large cohort of well-documented HIV-positive and HIV-negative persons using fungal internal transcribed spacer 2 (ITS2) amplification and sequencing and to identify clinical variables that modulate the oral mycobiome.

## RESULTS

We recently reported the distribution of secondary clinical variables for this cohort (30). The HIV-positive and -negative groups were recruited to achieve as much balance as possible regarding demographics, sexual activity, and oral health factors. Yet a number of secondary clinical variables were significantly different by HIV status, including race, illicit drug use, frequency of oral sex, dry mouth, past teeth cleanings, antibiotic usage, antifungal usage, oropharyngeal candidiasis (OPC), gingivitis, missing teeth, sampling site (clinic), and age (see Fig. S1 in the supplemental material). These interactions were considered in the previously reported bacterial community analyses and were also evaluated in this study of the mycobiome.

10.1128/mBio.00294-21.1 FIG S1 Balance for secondary clinical variables between the HIV-positive and -negative groups. Stacked bar graphs compare categorical variables for HIV^+(HAART)^ and HIV^−^ groups. Dichotomous categories are shown to the right of the labels. Significance was determined by Fisher’s exact test. Box and whisker plots of distributions for the HIV groups are shown for continuous variables. Significance was determined by Wilcoxon signed-rank test. Asterisks indicate levels of significance, as follows: *, *P* < 0.05; **, *P* < 0.01; ***, *P* < 0.001; ****, *P* < 0.0001. Group labels are HIV^−^ for HIV negative and HIV^+HAART^ for HIV positive under HAART. HIV+PostART, presence of HIV under HAART; OPC, oropharyngeal candidiasis; clinic; site of sampling; LSU, Louisiana State University; MCG, Medical College of Georgia. Reproduced with permission from Sci Rep. (30). Download FIG S1, TIF file, 0.8 MB. Copyright © 2021 Fidel et al. 2021 Fidel et al. https://creativecommons.org/licenses/by/4.0/ This content is distributed under the terms of the Creative Commons Attribution 4.0 International license.

**FIG 1:**
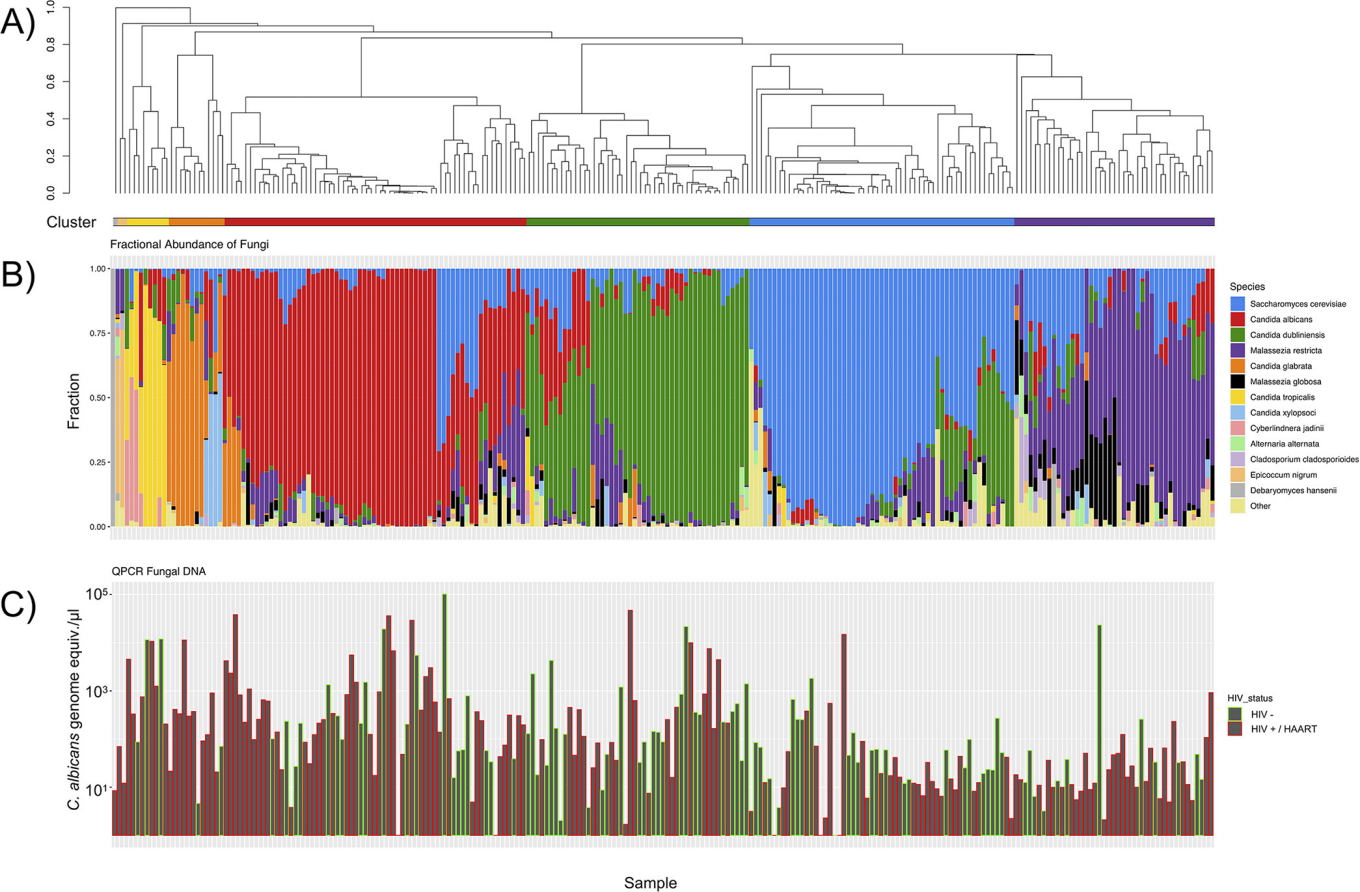
Clustering of oral mycobiome samples. (A) A dendrogram showing hierarchical clustering of fungal communities by Bray-Curtis dissimilarity using the unweighted pair group method with arithmetic mean. The tree is ordered with tighter clusters to the left. Clusters derived by cutting the tree into 8 clusters are shown by the colors on the bar below. (B) Stacked bar chart of relative abundances of species in the samples in the same order as in A, with the most abundant 13 species colored as shown. (C) Total fungal concentration for the samples, colored by HIV status.

Figure 1 illustrates the relative abundance of oral fungal communities in the cohort of 149 HIV-positive and 88 HIV-negative subjects. Individuals had an average of 12 species. There were 168 species identified within 12 dominant genera via sequencing of the ITS2 region of the rRNA gene repeat. Within the 12 genera, 1 to 3 species dominated the composition in any 1 individual (Fig. 1A). The stacked bar chart in Fig. 1B depicts the fractional species abundance for the 13 most abundant species for all samples sequenced. The samples were ordered based on the clustering represented in Fig. 1A, resulting in 4 major clusters dominated by Saccharomyces cerevisiae (57 samples), Candida albicans (65 samples), Candida dubliniensis (48 samples), and Malassezia restricta (43 samples). The tree was split by stratifying the data into 8 clusters, which was required to preserve the 4 major groups; the other 4 minor clusters were dominated by Debaryomyces hansenii (1 sample), Epicoccum nigrum (2 samples), Candida tropicalis (9 samples), and Candida glabrata (12 samples). Figure 1C shows the total fungal DNA as measured by quantitative PCR (qPCR) with samples in the same order as Fig. 1A and B. The bars are stratified by HIV status, revealing that the total fungal DNA varies by a large amount from sample to sample with HIV+/HAART samples found in all the major clusters.

In our earlier work, we reported results of *Candida* culture on these samples (30). To attempt to culture *Malassezia* species, 23 subjects who had a high relative abundance of *Malassezia* and low *Candida* species, and 13 subjects who had high *Candida* and low *Malassezia* species, were resampled and cultured on lipid-rich Chromagar and standard Chromagar with appropriate positive controls for each genus. Regardless of high or low abundance of *Malassezia* sp. via sequence analysis, none of the samples could cultivate *Malassezia* sp., whereas the 13 high *Candida* subjects were positive for *Candida* sp. on resampling.

Table 1 presents data that were partially reported in our previous publication (30) on the prevalence of *Candida* sp. by culture and compares it to the prevalence by sequencing. A total of 59% of HIV-negative and 63% of HIV-positive subjects had positive *Candida* cultures. Of those that were culture positive, the species included C. albicans (93%), followed by C. glabrata (24%), C. parapsilosis (7%), C. krusei (6%), C. dubliniensis (5%), C. tropicalis (4%), and others (7%), with mixed species and non-C. albicans exclusively identified in 13% and 4% of subjects, respectively. The species breakdown was similar for both HIV-negative and -positive subjects. Of note, Chromagar does not definitively differentiate C. albicans and C. dubliniensis. Hence, compared with the DNA sequence, C. albicans and C. dubliniensis culture results were disparate (Table 1). Supporting this dichotomy was the large overlap of subjects with both C. albicans and C. dubliniensis by DNA identification but not by culture. Overall, *Candida* species presence/abundance by individual and based on the DNA sequence correlated well with positive cultures/individual for the same species (Table 1). The exceptions were C. parapsilosis and C. krusei that, while detected by culture and present in the DNA sequence database, were not found by sequencing for reasons that are not clear.

**TABLE 1:** *Candida* species abundance/presence by DNA versus culture-positive results

Species	% subjects (*n*/total) positive by:	
	DNA sequence	Culture^*a*^
C. albicans*/*C. dubliniensis	77 (193/252)	60 (152/252)
C. albicans	62 (155/252)	57 (144/252)
C. dubliniensis	43 (108/252)	3 (8/252)
C. glabrata	23 (59/252)	15 (37/252)
C. tropicalis	14 (34/252)	2.45 (6/252)

a Culture was by Chromagar based on color reaction, as follows: C. albicans, dark green; C. dubliniensis, light green; C. glabrata, purple; and C. tropicalis, blue.

**FIG 2:**
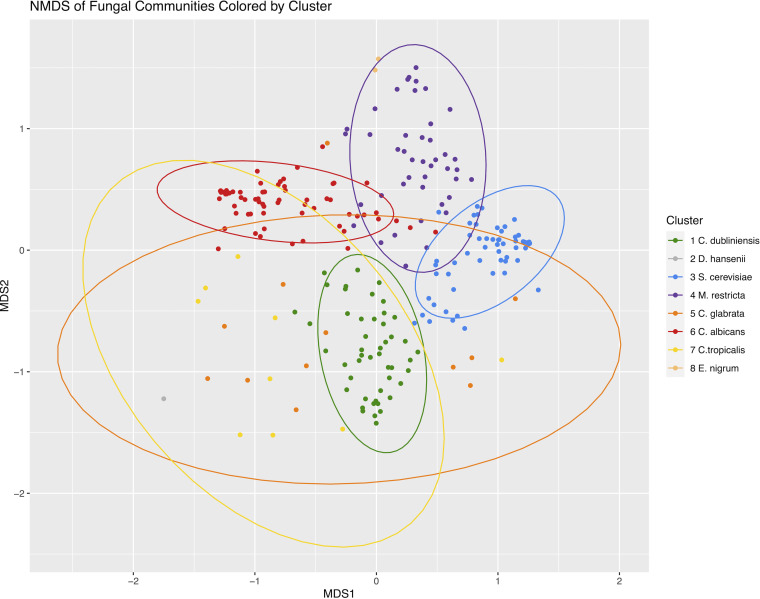
Nonmetric multidimensional scaling (NMDS) of fungal communities based on Bray-Curtis dissimilarities stratified by community clusters determined as in Fig. 1. Clusters are identified with the predominant fungi. Ellipses are 95% confidence areas of the group centroids. Clusters 2 and 8 do not have enough members to calculate an ellipse.

Figure 2 shows a nonmetric multidimensional scaling (NMDS) of the study samples based on Bray-Curtis dissimilarity of their fungal communities. The points are colored based on their clustering as in Fig. 1. As expected, the four major clusters appear as tight groups.

Multiple secondary variables were evaluated in samples for which information was available. Of the 24 variables in the analyses, 6 had a significant effect on the fungal community composition when considered individually, namely, caries index, missing teeth, age, HIV under HAART, clinic sampling site, and sex. Other variables having no significant effect included race, current cigarette smoking, dry mouth, gingivitis, periodontal disease, caries restoration, antimicrobials, floss frequency, brushing frequency, past cleanings, mouthwash usage, frequency of alcoholic beverage intake (daily/weekly), nonalcoholic beverage intake, diet, marijuana usage, illicit drugs, and frequency of oral sex.

**FIG 3:**
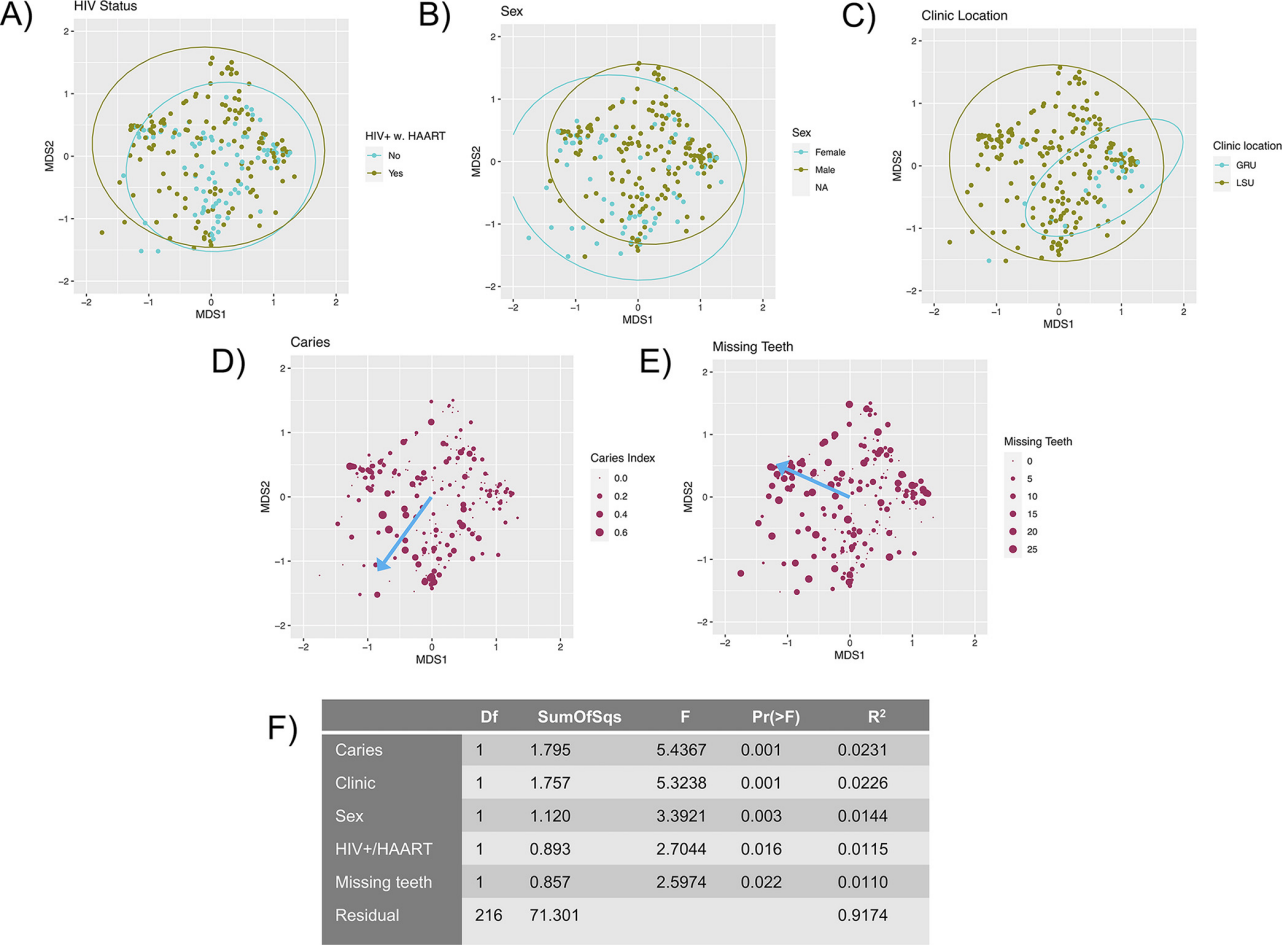
Effect of significant clinical variables on the fungal communities. (A to E) The identical NMDS ordination as in Fig. 2 (refer to Fig. 2 for the coordinates of the fungal community clusters on the graph). Ellipses indicate 95% confidence intervals of group centroids. The blue arrows indicate the direction of increase of ordinal variables determined by the envfit function. Directions of effects of categorical variables can be determined by the ellipse positions. (A) Points colored by HIV status. (B) Points colored by sex. (C) Points colored by the clinic location where they were collected. (D) Points sized by caries index, and arrow indicating the direction of increase. (E) Points sized by the number of missing teeth excluding third molars, and arrow indicating the direction of increase. (F) ANOVA table of a distance-based redundancy analysis of the marginal effects of the variables. Model variables were selected by an initial screening by PERMANOVA followed by a stepwise process based on adjusted R^2^ values.

To determine the marginal contributions of the six potentially confounding factors, a stepwise model selection using adjusted R^2^ and a constrained ordination method (distance-based redundancy analysis [dbRDA]) was used. This multivariate model identified five of the six variables as influencing the fungal community structure; age was excluded. Figure 3 shows the distribution of the five variables that remained in the resulting dbRDA model on the NMDS plot together with a table insert denoting the marginal effects of each variable expressed as R^2^ values with *P* values. It is notable that HIV-positive samples are shifted toward the C. albicans and *M. restricta* predominant clusters; samples from females, more missing teeth, and higher caries index are shifted toward the *Candida*-dominated clusters; and samples collected in the Medical College of Georgia clinic are shifted toward the C. dubliniensis and S. cerevisiae-dominated clusters. Within the HIV parameters, neither HIV load, CD4 cell count, nor HAART regimen (reverse transcriptase inhibitor [RTI] alone, RTI with the addition of integrase inhibitors, or RTI with the addition of nonnucleoside reverse transcriptase inhibitors [NNRTIs]) significantly affected the fungal community composition (data not shown).

**FIG 4:**
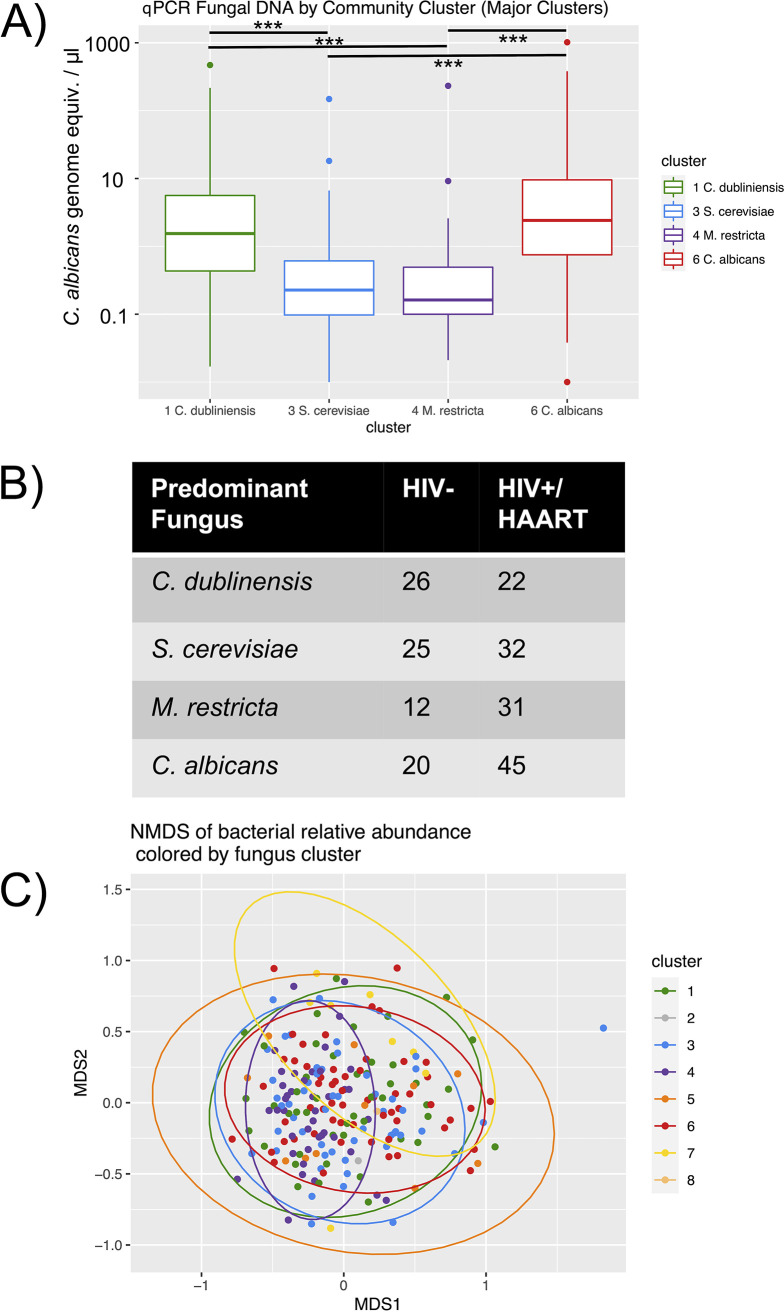
Differences between the four major fungal community clusters. (A) Total fungal content by qPCR versus fungal community cluster. A Kruskal-Wallis test of overall differences was highly significant (*P* = 2.4 × 10^−12^), and bars indicate significant pairwise Mann-Whitney Wilcoxon tests (***, *P* < 0.001). (B) A contingency table of HIV status versus fungal community cluster. *P* = 0.027 by Fisher’s exact test. (C) An NMDS of bacterial communities colored by the predominant fungal cluster with ellipses indicating 95% confidence intervals of the group centroid.

Further analyses of the major fungal clusters are shown in Fig. 4. When the total fungal DNA quantitated by qPCR is compared by fungal cluster (Fig. 4A), it is apparent that the two *Candida*-predominant clusters had higher fungal amounts than either the *M. restricta*- or S. cerevisiae-predominant clusters, and this result was confirmed by statistical testing (Kruskal-Wallis, *P* = 2.4 × 10^−12^; and *post hoc* Mann-Whitney Wilcoxon tests, 5.3 × 10^−6^ > *P* > 5.5 × 10^−9^ for significant tests, *P* > 0.28 for nonsignificant). Fig. 4B shows a contingency table for HIV status versus fungal cluster. As suggested by Fig. 3B, HIV+/HAART subjects more frequently than expected have either the *M. restricta* or C. albicans predominant fungal composition (*P* = 0.027, Fisher’s exact test). Fig. 4C shows the NMDS ordination of the bacterial communities, with colored sample data points representing the fungal composition clusters. Permutational multivariate analysis of variance (PERMANOVA) analysis indicated that there was an overall significant difference between the four clusters. Thus, pairwise PERMANOVAs were performed, which showed significant differences between the samples dominated by *M. restricta* and each of the other three clusters, but not between any of the other pairs. Significant PERMANOVA results can be caused by differences in beta dispersion, which was significantly lower for the *M. restricta* cluster (0.42) than the other 3 clusters (0.45 to 0.47; *P* = 0.04).

**FIG 5:**
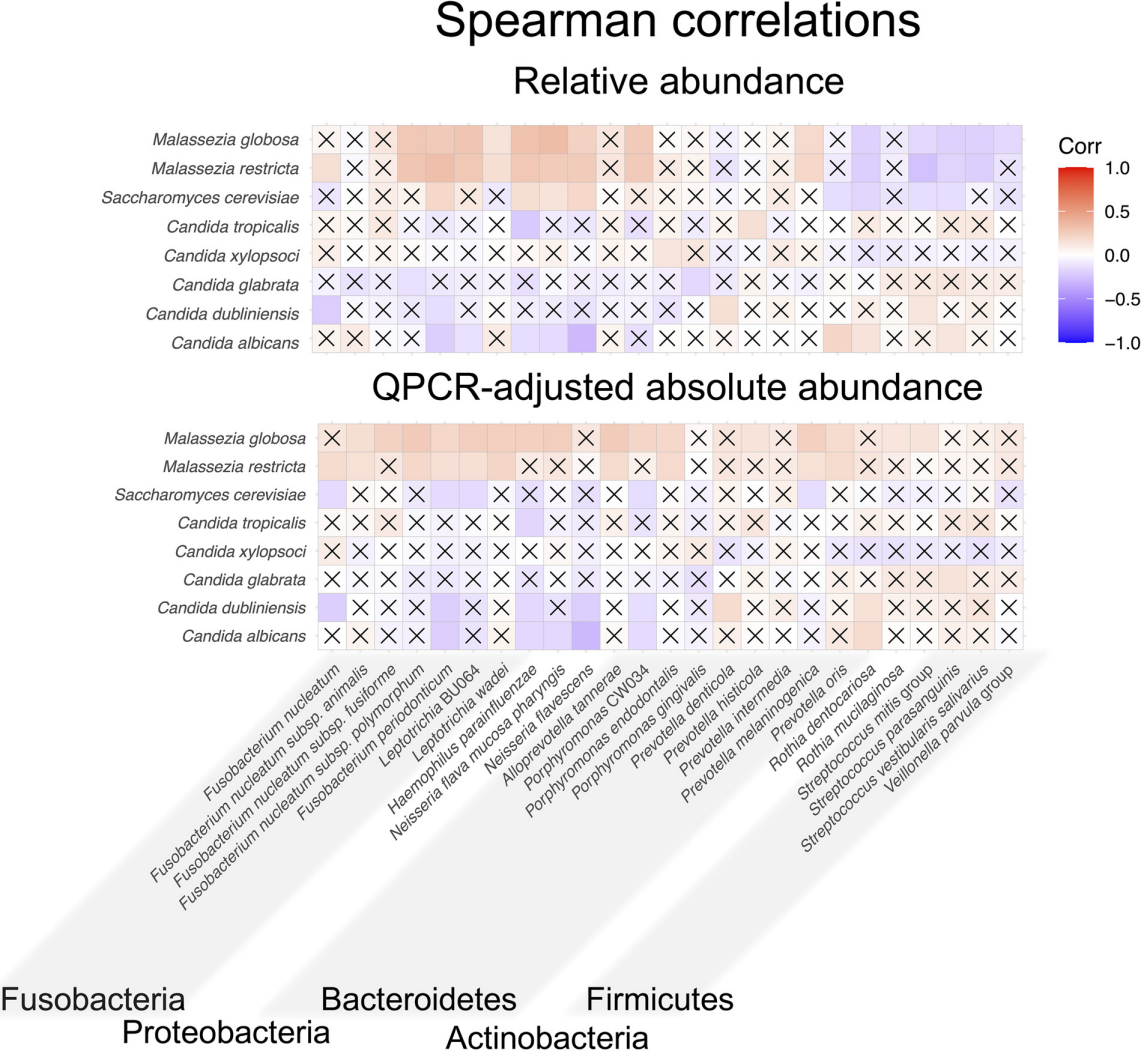
Correlation analyses of the most abundant fungal and bacterial species. Fungal and bacterial species that had a mean relative abundance of greater than 1% were selected, and the Spearman correlation over samples was calculated and is shown as on the scale. In the top panel, the correlation was determined based on relative abundance, while in the bottom panel it was based on qPCR-adjusted absolute abundance for both the fungus and bacteria. Interactions where the correlation was not statistically significantly different from zero are marked with Xs. The bacteria are grouped by phylum as indicated.

To determine associations between the fungal community composition and the bacterial community composition, Spearman correlation coefficients were calculated for fungal species and bacterial species that were more than 1% mean relative abundance. The calculations were done using both the relative abundance (Fig. 5A) or the absolute abundances that had been adjusted by qPCR measurements of total bacteria and total fungi (Fig. 5B). Results revealed that *Candida* species positively correlated with *Firmicutes* or *Actinobacteria* and negatively correlated with *Fusobacteri*a, *Proteobacteria*, and *Bacteroidetes*.

## DISCUSSION

We previously reported on the oral bacterial community in this present cohort using whole-mouth gargle/rinse sampling (30). The primary conclusions were that multiple clinical factors have a significant influence on the oral bacterial community composition and diversity, including the presence of HIV under HAART. The impact of HIV/HAART, however, was much less than expected. In this study, the mycobiome was considered and assessed similarly for composition, diversity, and effect of clinical variables.

In contrast to the number of bacterial species identified in any 1 individual (average of 190 of ∼600 possible, 32%) (30), the fungal species per individual were much fewer (average of 12 of ∼170 found in any sample, 7%) and largely dominated by 1 to 3 primary species in greatest abundance. There are obviously fewer fungal species overall, as expected, but the relative abundance being restricted to so few species is a major distinction to the oral bacterial microbiome. We identify four major clusters of fungal communities, with the predominant species being Saccharomyces cerevisiae, Candida albicans, Candida dubliniensis, and *Malassezia restricta*, together with four minor clusters dominated by other species.

Although samples from the four major clusters were found in both the HIV-negative and HIV-positive/HAART groups, there were significant deviations from expected ratios, including a statistically higher association of the C. dubliniensis-predominant cluster with the HIV-negative group. This finding is in contrast to previous culture data showing C. dubliniensis is more prevalent in HIV-positive individuals (31). This apparent discrepancy could be due to differences in detection by culture versus sequencing, effects of other clinical variables that are not balanced between the groups, differences between the American and Irish study populations, or changes in HIV treatments since the 1990s. Of note, although the detection of C. dubliniensis by culture may not have been definitive, there was no difference in what was considered C. dubliniensis via culture regarding asymptomatic colonization for both groups in our cohort and only one case of OPC involving C. dubliniensis in the HIV-positive group.

It is unclear what factors are responsible for relative species dominance, but they could include the competitive nature of the fungal species themselves, bacterial species, or the oral environment. Hong et al. recently reported two major clusters of salivary fungal communities dominated by *Candida* and *Malassezia* species, but did not identify two of the more common *Candida* species or a *Saccharomyces* sp.-dominated population (15). Possible explanations for the discrepancy in these findings could be differences in the study populations, the region sequenced (ITS1 versus ITS2), or sampling methods.

Our study identified 12 fungal genera and that oral fungal communities tended to be dominated by *Candida*, *Saccharomyces*, and *Malassezia* species. Other genera included *Cladosporium*, *Alternaria*, *Epicoccum*, *Aspergillus*, *Fusarium*, *Filobasidium*, *Debaryomyces*, *Cyberlindnera*, *and Aureobasidium*. While there have been only a limited number of studies assessing the human oral mycobiome, all report a similar set of genera with *Candida* sp. being prominent and with a variable abundance of other fungal genera (15–19, 32). The *Candida* species identified were consistent with those often cultured from the oral cavity and considered normal flora and opportunistic pathogens (33), including C. albicans, C. dubliniensis, C. glabrata, and C. tropicalis. The exception was Candida xylopsoci that is rarely identified in culture. For *Malassezia* sp., we and others identified a predominant abundance of *M. restricta* and M. globosa (21). Taken together, these data show a clear trend for a signature core oral mycobiome.

These data, however, are not without limitations. Regarding the use of qPCR to quantitate the bacterial and fungal communities, we used primers within the rRNA genes and standardized the values to genomic DNA from one species. The amplicon sequencing data were then used to partition the relative contributions of the different species. The use of one standard allows results to be compared between runs. We recognize possible sources of error in this approach, including amplification biases, variable genome sizes, and variable copy numbers of the rRNA genes. For eukaryotes such as fungi, the rRNA copy number may also vary between strains of the same species or by environmental conditions (34). The qPCR measurements therefore should be interpreted cautiously.

The observation that clusters of samples dominated by *Saccharomyces* and *Malassezia* species had lower total fungal content by qPCR than clusters of samples dominated by *Candida* species raised the possibility that the *Malassezia* and *Saccharomyces* species might be nonviable organisms present in small amounts. S. cerevisiae is of course present in many food and beverage products, and *Malassezia* species are known to be present on skin (35). Thus, both might enter the oral cavity transiently. A recent study of fungal content of human stool by ITS2 sequencing found that S. cerevisiae was present with a normal diet but was not found with a highly controlled diet lacking the organism (36). *Malassezia* species typically do not make fatty acids and require high concentrations of lipids in culture, generally much higher than the lipid content of saliva (37–40). Attempts to address the viability issue by resampling several patients dominated by *Malassezia* sp. and culturing the rinse samples on lipid-rich Chromagar specific for *Malassezia* species showed no growth. In contrast, growth was observed on standard Chromagar for several individuals dominated by *Candida* species who were resampled. Hence, it would not appear that the *Malassezia* species identified were viable. However, a caveat is that the absolute fungal abundance tended to be low in samples dominated by *Malassezia* sp., and the samples cultured were not those but rather ones from subjects showing high relative abundance. Another laboratory that similarly attempted to cultivate *Malassezia* sp. from oral samples also showed largely negative results. The lone species reported to be cultured in that study was Malassezia sympodialis that was not in high abundance by sequence analysis (21). Further research is needed on this interesting issue of viability.

Of the 24 potential influential clinical variables (18 categorical, 6 continuous), only 5 had a significant impact on the oral mycobiome as a whole. These variables included the HIV under HAART, geographical site of sampling, sex, missing teeth, and caries index (determined by decayed and restored surfaces). All were also significant variables in the bacterial community analyses (30) and thus seem to have an influence on both branches of the oral microbial community. Notably, the R^2^ values, similarly to the bacterial community analyses, indicate that all the clinical variables measured here explain only a small percentage of the total variation in microbial community composition. Of note, though, each of the significant variables affected the mycobiome even in a strict model designed to consider only marginal effects isolated from those of the others. Clinic site was also shown to impact the bacterial microbiome. Our favored explanation for this variable, similar to the bacterial community analyses (30), is that small differences in the techniques for the collection of specimens contributed to the differences observed, although geographic effects cannot be ruled out completely. In the bacterial community analysis, 10 variables were significantly influential, including HIV/HAART (30). Recognizing that HIV under HAART had only a modest influence on the oral bacterial community, it was not surprising that HIV/HAART had a similarly modest influence on the mycobiome. Yet the influence of HIV/HAART on the entire microbiome may be indicative of clinical significance. The smaller number of variables influencing the mycobiome than those of the bacterial community may be due to the relatively small number of species identified in any one individual and the tendency for samples to be dominated by so few fungal species.

The influence of HIV on the oral microbiome has now been evaluated in several cohorts. Like the majority of bacterial community analyses (2, 11, 12, 30), those that evaluated the mycobiome also report a modest impact of HIV (10, 23–25), although differences in dominant genera have been shown (i.e., *Epicoccum* and *Alternaria* more in HIV-positive versus *Pichia* and *Fusarium* more in HIV-negative individuals) (10). The latter may be due to the diversity of sampling that included palatine tonsil, oral rinse, or saliva. It would be interesting to compare subgingival samples to whole-mouth samples for effects of HIV or composition/diversity. Like our cohort, most studies evaluated well-controlled HIV-positive adults on HAART HIV (10, 23–25). However, none have observed any notable effect of biomarkers of immune stimulation/dysfunction (i.e., CD4 cell number/HIV load) on mycobiome diversity (22). Likewise, our bacterial community analyses failed to show any differences between any specific HAART regimens and time on HAART (41). Yet studies identifying specific effects of HAART alone (bacterial microbiome and mycobiome) are still needed. Accordingly, we are currently conducting longitudinal bacterial community and mycobiome analyses comparing HIV-positive subjects and also HIV-negative pre-exposure prophylaxis (PrEP) persons pre-HAART and at several time points after initiating therapy.

We observed significant interactions between bacterial and fungal communities by two methods. One method was to use the 4 major clusters of samples by fungal community to examine if we could detect differences in bacterial communities. In that analysis, the *Malassezia restricta*-dominated cluster showed significant differences from the other 3 clusters. As another method of studying bacterial-fungal interactions, we calculated correlations between the most abundant bacterial and fungal species. *Fusobacteria*, *Proteobacteria*, and most *Bacteroidetes* sp. were positively correlated with *Malassezia* sp. and negatively correlated with *Candida* sp. using either relative or absolute abundances. *Actinobacteria* and *Firmicutes* were positively correlated with *Candida* species, and their correlation with *Malassezia* sp. depended on which abundance measurement was used. *Firmicutes* and *Actinobacteria* are the major Gram-positive phyla, although *Veillonella* spp. are Gram negative, as a genus of the *Negativicutes* class. Hong et al. show a clear association of oral *Candida* sp. with Gram-positive aciduric bacteria and an association with dental caries, while oral *Malassezia* sp. showed an association with inflammophilic bacteria (15). Another group reported associations between early and advanced caries, with *Candida* species in the oral mycobiome together with *Malassezia* species capable of inhibiting caries-associated Gram-positive bacteria (21). Still, others show negative correlations between *Candida* and *Neisseria* sp. (Gram negative) in the microbiome of palatine tonsil of HIV-positive persons (24) and clear evidence of several fungal and bacterial pairings in the microbiome, including in HIV-positive persons (23). If one takes into account the influence of *Candida* culture positivity as a variable on the bacterial community (30), together with the physical predilection of some Gram-positive bacteria for C. albicans hyphae, an association between Gram-positive bacteria and *Candida* species may be predicted. The observed correlations could be due to shared nutritional requirements, especially considering the association of high sugar diets with caries. Nevertheless, these observed interactions between bacteria and fungi in the oral cavity are interesting and require more in-depth analyses.

In conclusion, after analyses of the oral mycobiome in a large cohort of HIV-negative and -positive persons, we have identified several distinctions to the oral bacterial community, namely, dominance by few fungal genera/species and fewer significantly influencing clinical variables. Overall, the mycobiome appears quite stable, although changes occur with age. A modest yet significant effect of HIV with HAART, caries status, and missing teeth on both the oral mycobiome and bacterial community implies that these factors impact the whole oral microbiome. We also identified an interesting association between phylum-level groups of bacteria with *Candida* and non-*Candida* genera, respectively. This study represents one of the first in-depth analysis of the oral mycobiome in a large human cohort, including those with HIV disease. These results together with those reported previously may direct therapeutics and provide explanations for other pathological questions in oral health/disease.

## MATERIALS AND METHODS

### Clinical methods.

Recruitment, clinical data collection, and sample collection were carried out as described (30).

### Asymptomatic oral yeast colonization and diagnosis of OPC.

Yeast were identified by plating samples onto Chromagar (CHROMagar Microbiology) and incubating them for 48 h at 37°C to observe colony growth. Initial species identification was assessed according to colony color. All rinse samples were screened for asymptomatic yeast colonization by plating 50 μl of each sample. Some patients were resampled and cultured for *Malassezia* sp. on lipid-rich Chromagar as well again on standard Chromagar. *Malassezia restricta* purchased from American Type Culture Collection (ATCC) served as the positive control. For subjects with OPC (*n* = 16), the lesions were dominated by C. albicans (94%) as expected, although mixed species were also identified 25% of the time and included C. glabrata, C. krusei, and/or C. tropicalis. Only one case was identified as non-C. albicans exclusively.

### DNA extraction and sequencing.

DNA was prepared from oral rinse samples with slight modifications of the QIAamp DNA blood minikit (Qiagen, USA) (42) Initially, 300 μl of the oral rinse was added to 200 μl of ATL buffer and treated with proteinase K for 2 h at 56°C. The samples were then homogenized with 0.25 g of 0.5-mm glass beads in a Mini-Beadbeater-16 (BioSpec Products, USA), before proceeding with the kit instructions. Elutions were performed with 2 × 30 μl of buffer AE heated to 42°C.

Sequencing libraries were prepared by a variant of the Illumina 16S protocol (41) modified to include fungal primer sequences specific for the fungal ITS2 region as well as for automation, lower input concentration of fungal DNA, and inclusion of additional index sequences. The primers used were 5.8S_Fa (TCG TCG GCA GCG TCA GAT GTG TAT AAG AGA CAG TCG ATG AAG ARC GCA GC) and 28S_Ra (GTC TCG TGG GCT CGG AGA TGT GTA TAA GAG ACA GTA TGC TTA AGT TCA GCG GGT A), with the gene-specific portions of the sequences underlined. The primers contained a phosphorothioate bond at the 3′ end. The liquid handling was performed in a Biomek 4000 instrument (Beckman Coulter). In the procedure, 5.8S and 28S gene-specific primers with 5′ tails were used in an initial amplicon PCR, while dual indices and specific adapters were added by a second index PCR with a small number of cycles. Oral rinse DNA was adjusted to 5-ng/μl concentration and 2 μl of DNA was amplified in a 25-μl reaction with Accuprime *Taq* high-fidelity DNA polymerase (ThermoFisher, USA). The cycling conditions were initial denaturation at 94°C for 2 min; 35 cycles of 94°C for 30 sec, 55°C for 30 sec, and 68°C for 1 min; and a final extension of 72°C for 5 min. Amplicons were purified with 20-μl AMPure XP magnetic beads (Beckman Coulter) and used as the template in the index PCR which used identical times and temperatures but for only 8 cycles. Sequencing was performed on the MiSeq platform with 2 × 300-base pair reads.

### Sequence data processing.

Forward and reverse sequence reads were combined using mothur *make.contigs*, mothur *screen.seqs* was used to select fragments larger than 150 bases with less than 10 ambiguous bases, and mothur *trim.seqs* was used to remove primer sequences (43). Sequences derived from read pairs with mean base Phred quality less than 28 were removed using a script based on Biopython (44). Sequences were aligned against the ISHAM ITS database (45) using BLASTN, followed by a php script to rescore the percent identity (avoiding counting ambiguous matches as mismatches). Finally, the best matches over 98% identity were selected to assign taxonomy to the sequences to the species level. The data were loaded into a MySQL database with sample information via a shell script.

### qPCR for total bacterial and fungal DNA.

The amount of bacterial DNA was quantitated with a Bio-Rad iCycler real-time detection system. One microliter of DNA was included in a 20-μl reaction with 1× SsoFast EvaGreen Supermix (Bio-Rad) and primers Eub338F (ACT CCT ACG GGA GGC AGC AG) and Eub518R (ATT ACC GCG GCT GCT GG). The thermal steps were 98°C for 2 min followed by 45 cycles of 98°C for 5 sec and 64°C for 5 sec. Purified Porphyromonas gingivalis genomic DNA was used as a standard.

Fungal DNA was measured by qPCR of the ITS2 region. The primers used were 5.8s-F (TCG ATG AAG ARC GCA GC) and the 28S reverse primer that was used for sequencing. The cycling conditions and instrument settings were the same as used for the bacterial qPCR. Purified Candida albicans genomic DNA was used as the standard.

### Statistical analysis.

Clinical data were collected and calculated as described (30).

Fungal community samples were clustered by the unweighted pair group method with arithmetic mean using the hclust function in base R (“average” method) (46). Clusters were formed with the cutree function with k = 8, which was the minimum number needed to preserve the four major visually impactful groups. Nonmetric multidimensional scaling of Bray-Curtis dissimilarities was performed using the metaMDS function from the vegan package in R (47). The contributions of clinical variables to fungal community composition were determined with a nonparametric permutation-based analysis of variance (PERMANOVA), implemented by the adonis2 function in vegan. The direction of the effect of continuous clinical variables in the NMDS was modeled using the envfit function in vegan, and the magnitude was scaled to the effect size (R^2^) determined by PERMANOVA. Spearman correlations were determined with the rcorr function of the Hmisc package in R (48). Significance tests were performed with the kruskal.test, wilcox.test, and fisher.test functions of R.

Distance-based redundancy analysis (db-RDA) was done using capscale (vegan). Variables were included in a stepwise db-RDA model selection process (ordiR2step, vegan). The marginal significance of the remaining variables was tested by a permutation-based ANOVA for constrained ordinations (anova.cca, vegan). Only the clinical variables that were significant contributors to the variation in microbial community composition after adjusting for multiple comparisons (49) were entered into the model selection for the db-RDA ordination. Code used in the statistical analyses was deposited at the OSU code repository online at https://code.osu.edu/beall-3/hiv-mycobiome.git.

### Data availability.

The sequence files generated in this study have been deposited in the NCBI Sequence Read Archive (18), associated with BioProject PRJNA530161.
