# Supplementary figures and images for: Effect of HIV/HAART and Other Clinical Variables on the Oral Mycobiome Using Multivariate Analyses
Source: mBio. 2021 Mar 23;12(2):e00294-21. doi: 10.1128/mBio.00294-21 (PMC8092233; doi:10.1128/mBio.00294-21)

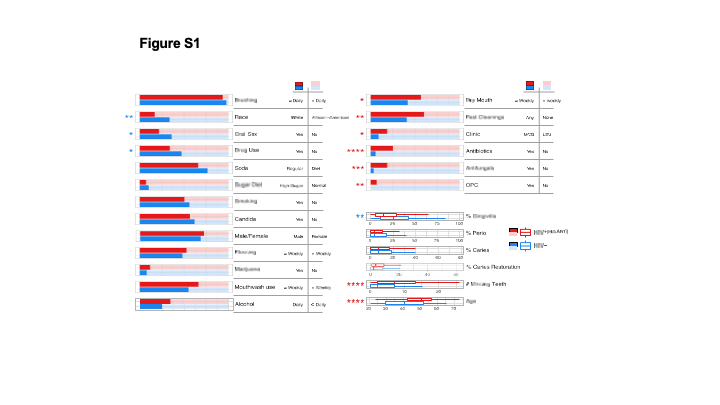

Supplement: FIG S1 [file mBio.00294-21-sf001.tif]
